# Study on the relation of the solar coronal rotation with magnetic field structures

**DOI:** 10.1038/s41598-023-48447-0

**Published:** 2023-11-30

**Authors:** N. B. Xiang, X. H. Zhao, L. H. Deng, F. Y. Li, S. Zheng

**Affiliations:** 1 Yunnan Observatories, Chinese Academy of Sciences, Kunming, 650011 China; 2 State Key Laboratory of Space Weather, National Space Science Center, Chinese Academy of Sciences, Beijing, 100190 China; 3 School of Mathematics and Computer Science, Yunnan Minzu University, Kunming, 650504 China; 4 Institute of Optics and Electronics, Chinese Academy of Sciences, Chengdu, 610209 China; 5 Key Laboratory of Modern Astronomy and Astrophysics (Nanjing University)-Ministry of Education, Nanjing, 210093 China; 6 The Key Laboratory on Adaptive Optics, Institute of Optics and Electronics, Chinese Academy of Sciences, Shuangliu, P.O. Box 350, Chengdu, 610209 Sichuan China; 7 College of Science, China Three Gorges University, Yichang, 443000 China

**Keywords:** Solar physics, Solar physics

## Abstract

Daily solar spectral irradiances (SSIs) at the spectral intervals 1–40, 116–264 and 950–1600 nm and four categories of solar small-scale magnetic elements ($MF_{no}$, $MF_{anti}$, $MF_{tran}$ and $MF_{in}$) are used to study the temporal variation of coronal rotation and investigate the relation of the coronal rotation with magnetic field structures through continuous wavelet transform and Pearson correlation analysis. The results reveal the contributions of different magnetic structures to the temporal variation of the rotation for the coronal atmosphere during different phases of the solar cycle. During the solar maximum, the temporal variation of rotation for the coronal plasma atmosphere is mainly dominated by the small-scale magnetic elements of $MF_{anti}$; whereas during the epochs of the relatively weak solar activity, it is controlled by the joint effect of the small-scale magnetic elements of both $MF_{anti}$ and $MF_{in}$. The weaker the solar activity, the stronger the effect of $MF_{in}$ would be. Furthermore, this study presents an explanation for the inconsistent results for the coronal rotation issue among the previous studies, and also reveals the reason why the coronal atmosphere rotates faster than the lower photosphere.

## Introduction

The study on solar coronal rotation is an interesting and challenging research topic since the results derived from different studies could be completely different, even contradictory to each other. These include the synodic (sidereal) rotation period, as well as its radial and temporal variation, the characterization of differential rotation or near-rigid rotation, and the relationship of the coronal rotation period with the solar cycle phase, and so on^1–9^. Moreover, the corona is optically thin, there are virtually no solar long-life structures (tracers) which can present the prominent rotating tracer features in terms of the spatial and temporal extent in the corona^7,8,10^.Thus, it is impossible to study the rotation of the solar corona in the same way as using tracers such as sunspots to investigate the rotation accurately in the photosphere. Meanwhile, it has still been very difficult to measure the coronal magnetic field accurately until now^2,7,11^. As a consequence, the solar coronal rotation is still lacking conclusive results, and in fact the study on this issue is an open topic so far.

Generally, the research on the coronal rotation mainly used one or several spectral lines formed in the corona, such as X-ray, ultraviolet rays and radio emissions^7,8,12–15^. For instance, high-resolution observational studies have suggested that the solar corona is rotating differentially, even the differential rotation rate of the corona is consistent with latitudinal rotation profile of the underlying photospheric magnetic field^12,14,16,17^. However, Giordano & Mancuso^5^ suggested the ultraviolet coronal rotates less differentially than the photosphere , which was obtained by analysis the two ultraviolet emission lines of the O vi 1032 Å and H i Ly$\alpha$ 1216 Å. Similar results also can be found in the rotation of radio corona^18^ and soft X-ray corona^19^. Especially, Mancuso et al.^8^ used the five different ultraviolet spectral lines of O vi 1032 Å, O vi 1037 Å, Si xii 499 Å, Si xii 521 Å and H i Ly$\alpha$ 1216 Å to investigate the coronal rotation. This study shown that the rotation of the ultraviolet corona exhibits the quasi-rigid rotation at mid and high latitudes. Apart from the impact of quality of observational data, these different results may be due to the different mathematical methods and observational data (spectral lines formed at different altitudes in the corona) during different time intervals, which were adopted in these studies. Actually, relying solely on a few spectral lines formed in the corona during some certain time intervals cannot yield conclusive results about differential rotation of the corona. Moreover, the existing observational data do not allow for a detailed study of the temporal evolution of the differential rotation of the corona, which may yield convincing conclusions.

On the other hand, some special spectra lines and coronal activity indicators have been continuously observed for decades, which can be used to investigate the temporal variation of coronal rotation from a global point of view. A long time series of solar radio flux at the 10.7-cm wavelength during 1947–2009 was used to study the long-term variations of the coronal rotation in Li et al.^6^. This study indicated a weak decreasing trend in the rotation cycle length of solar corona, but such secular trend is no 11-year Schwabe cycle of statistical significance. Similarly, Xie et al.^20^ and Deng et al.^11^ used daily 10.7-cm solar radio emission flux for the time interval from 1947 to 2014 and the modified coronal index during the time interval of 1939 to 2019, respectively, to study the temporal variation of corona rotation. Both studies also discovered a decreasing trend in the rotation periods during the considered time. Furthermore, these two studies further identified several significant periods in the variations of the rotation cycle length of solar corona on timescales of 3 to 11 years. They suggested that the solar cycle variation of the coronal rotation is associated with the 11-year Schwabe cycle. However, this correlation between the variation of coronal rotation and the Schwabe solar cycle is inconsistent with the findings reported by Chandra & Vats^13^ and Li et al.^6^. Edwards et al.^16^ used a time series of tomographical maps from 2007 to 2020 to show an abrupt decrease of equatorial rotation rate of the solar corona in 2009, followed by a return to faster rotation in 2017. Since 2003 February 25, the SORCE satellite has continuously measured the daily solar spectral irradiances (SSIs) which are at 985 disjunct bands of spectral intervals 1–40 nm and 116–2416 nm. Using these daily observational records, Li et al.^21^ investigated the rotation of solar corona and photosphere by applying the Lomb-Scargle periodogram method. However, due to the limitations of the Lomb-Scargle periodogram method, the research results in this study only represented a mean result of the rotation of the corona and photosphere during 2003–2017, and the temporal variation of solar coronal rotation was ignored.

It is widely agreed that the coronal rotation period varies with time, and is modulated by some types of solar magnetic activity. However, there is currently no convincing research to address the magnetic field structures responsible for the temporal variation of coronal rotation. With the improvement of observation technology, high-resolution observations have shown that the solar surface is covered by ubiquitous small-scale isolated magnetic elements with a resolution of a dozen arcseconds or less, such as ephemeral regions, network and intra-network magnetic fields^22^. Ephemeral regions refer to small-scale emerging bipoles^23^. At the boundaries of supergranulation, the stronger magnetic elements are network elements, while within the supergranulation cells, the smaller and weaker elements are described as intra-network elements^24,25^. The 5-minute averaged full-disk magnetograms observed by the Michelson Doppler Imager on board the Solar and Heliospheric Observatory (MDI/SOHO) were used by Jin et al.^26^ to analyze the cyclic behavior of solar small-scale magnetic elements. The results indicated that the solar magnetic elements can be divided into large-scale magnetic elements in active regions and small-scale magnetic elements in quiet regions according to the flux per element. The ubiquitous small-scale magnetic elements are derived from several sources: debris of decayed active regions, small-scale flux emergence in the ephemeral regions, coalescence of intra-network elements, and products of dynamic interaction among different scales of magnetic activities^22,26^. For the ubiquitous small-scale magnetic elements in solar cycle 23, the millions of magnetic elements could be further classified into four categories: (1) those elements with magnetic flux in the range of $(1.5 - 2.9)\times 10^{18}$ Mx show basically no correlation with the sunspot cycle (referred to as $MF_{no}$); (2) network elements whose magnetic flux is in the range of $(2.9 - 32.0)\times 10^{18}$ Mx are anti-correlation with the sunspot cycle (referred to as $MF_{anti}$); (3) magnetic elements covering the flux range of $(3.20 - 4.27)\times 10^{19}$ represent a transition from anti-correlation to correlation with the sunspot cycle (referred to as $MF_{tran}$); (4) those elements falling in the range of $(4.27 - 38.01)\times 10^{19}$ Mx show in-phase with sunspot cycle (referred to as $MF_{in}$). Moreover, the authors further indicated that in solar cycle 23, the $MF_{no}$, $MF_{anti}$, $MF_{tran}$ and $MF_{in}$ accounted for 0.58%, 77.19%, 6.59% and 15.65% of the total number of small-scale magnetic elements, respectively, or contributed 0.05%, 37.40%, 9.08% and 53.46% of the total magnetic flux of network elements, correspondingly^26,27^. The classification of solar small-scale magnetic elements has enabled the investigation of the role of various magnetic elements in the coronal rotation of the Sun.

It is known that SSIs of the wavelengths shorter than 40 nm form in the solar corona, SSIs at the spectral intervals 40–264 nm form in the solar corona and transition region, and SSIs whose wavelengths are longer than 950 nm but shorter than 1600 nm form at the underlying photosphere^21,28–31^. The daily SSIs at the spectral intervals 1–40, 116-–264 and 950–1600 nm, along with the four categories of small-scale magnetic elements, are used to study the following two topics: 1. Analyze and compare the temporal variation of the rotation for the corona and the solar photosphere during long-term time interval, and then present an explanation for the inconsistent results for the coronal rotation problem among the previous studies; 2. Investigate the relation of the coronal rotation with magnetic field structures, and further identify which types of magnetic elements are responsible for the temporal variation of coronal rotation.

## Data and methods

**Figure 1:**
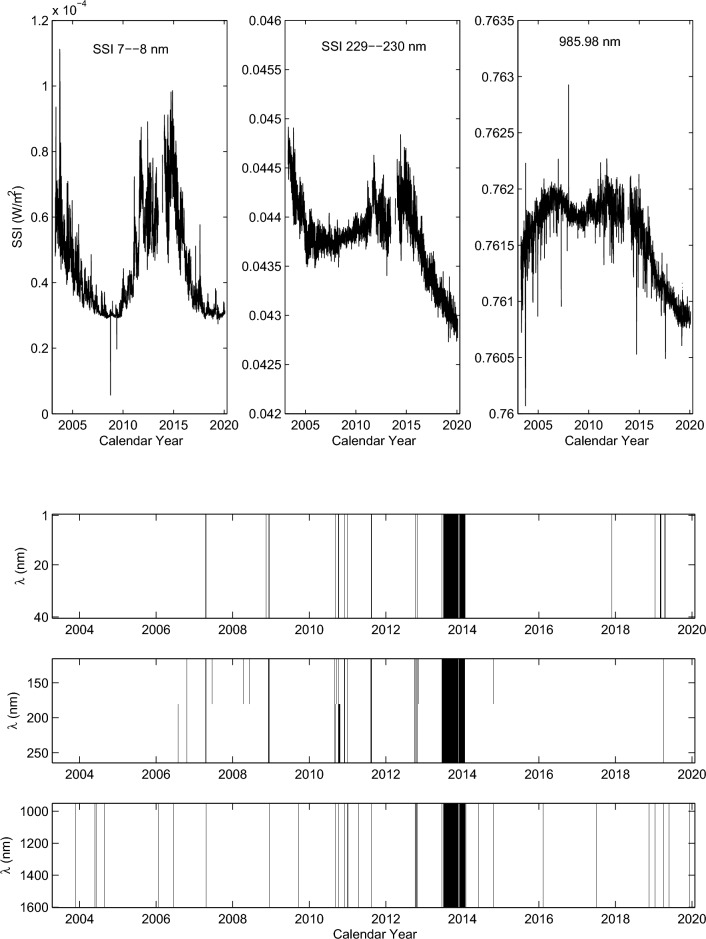
Top panel: Daily SSIs at three randomly selected bands from 2003 April 14 to 2020 February 25. Bottom panel: the vertical black lines (regions) indicate that the SSIs have no observed values in the corresponding time intervals.

Daily SSIs at the spectra intervals 1–40 nm and 116–2416 nm have been continuously measured by the SORCE satellite since 2003 February 25, but not all SSIs were measured from this day, even some SSIs were not measured until more than a month later. In order to study the temporal variation of coronal rotation and further address the magnetic field structures that are responsible for temporal variation of coronal rotation, we select the daily SSIs at the spectral intervals 1–40, 116–264 and 950–1600 nm during time interval of 2003 April 14 to 2020 February 25. These SSIs can be downloaded from web site http://lasp.colorado.edu/home/sorce/data/. Daily SSIs at three randomly selected bands during time interval considered are shown in the top panel of Fig. 1. In the bottom panel of Fig. 1, vertical black lines (regions) indicate all SSIs without observed values in the corresponding time intervals. For SSIs at the spectral intervals 1–40 and 116–264 nm, the span of a band is 1 nm, and there are a total of 189 spectral bands. For SSIs at the spectral intervals 950-1600 nm, the span of spectral bands is in the range of 4.67 to 5.5 nm, a total of 123 spectral bands are used. We use the wavelength (L nm) to replace “spectral band” in the figure, which actually is the middle value of a spectral band. As this figure shows, continuous no observed values of SSIs mainly occurred in the second half of 2013 to the first half of 2014.

The wavelet analysis can reveal the dominant periods at different timescales and localized oscillatory feature of a one-dimensional time series through power spectra analysis, which decomposes a time series into two-dimensional time-frequency space^32–35^. Moreover, the local wavelet power spectra can indicate the detailed information of the time-frequency components of the analyzed data, enabling the determination of frequency components at a certain point in time within the analyzed time series. Based on the local wavelet power spectra, the local highest spectral power peaks can be used to identify the characteristic periods of the analyzed time series at a certain time. As a consequence, the wavelet analysis is very suitable for studying the temporal evolution of solar coronal rotation in this study. Generally, three wavelet basis functions, the Morlet wavelet, Paul wavelet and DOG wavelet can be used as mother wavelet in the process of the continuous wavelet transform, but only the Morlet wavelet can be used for feature extraction, since it can provide a reasonable localization in both time and frequency^32,34^. Thus, it is a logical and wise choice to use the Morlet wavelet in this study. The Morlet wave can be defined as

1 $$\begin{aligned} \Psi _{0}(\eta )=\pi ^{-1/4}e^{i\omega _{0}\eta }e^{-\eta ^{2}/2} \end{aligned}$$

where $\eta$ is dimensionless time and $\omega _{0}$ is dimensionless frequency. In the process of the continuous wavelet transform, the number of oscillations in the mother wavelet is controlled by $\omega _{0}$, and so the time and frequency resolution of the corresponding wavelet transform must be affected by the value of $\omega _{0}$^36^. Furthermore, smaller values of $\omega _{0}$ chosen in the continuous wavelet transform correspond to a better time resolution, but larger values of $\omega _{0}$ can obtain a higher frequency resolution, which indicates a more accurate resolution of the period^33,37^. In this study, we try different values of $\omega _{0}$, and find that dimensionless frequency $\omega _{0}=12$ is a good choice, since it can reasonably give both time and frequency resolution. Finally, the statistical significance test of wavelet power is assessed through the assumption that the noise has a red spectrum.

## Temporal variation in the rotation of the corona and the underlying photosphere

the whole measured daily SSIs span 6161 days, but the number of no-data days during the time interval we considered is about 340-380 days, accounting for about 5.5–6.2%. However, as Fig. 1 shows, the vertical black lines (regions) in bottom panel, which represent the SSIs without observed values in the corresponding time intervals, indicate that the continuous data gaps mainly concentrated from 2013 July 16 to 2014 February 25, lasting for 225 days. We cut out this continuous data gaps from the entire time interval considered, and cut these time series of SSIs into two time intervals, namely, from 2003 April 14 to 2013 July 15, and from 2014 February 26 to 2020 February 25. The total number of no-data days of the SSIs during the two time intervals we considered is about 120–160 days, accounting for only 2.0–2.7%. Moreover, these data gaps are randomly distributed within the two time intervals considered, and the continuous data gaps in these time series of SSIs are generally shorter than 5 days, which is much shorter than the timescales of a rotation period. Thus, the data gaps have little impact on the application of the continuous wavelet transform (CWT) for analyzing solar coronal rotation after data gap interpolation. The method of the linear interpolation is utilized to interpolate the value when the SSIs have no observed value on a certain day.

**Figure 2:**
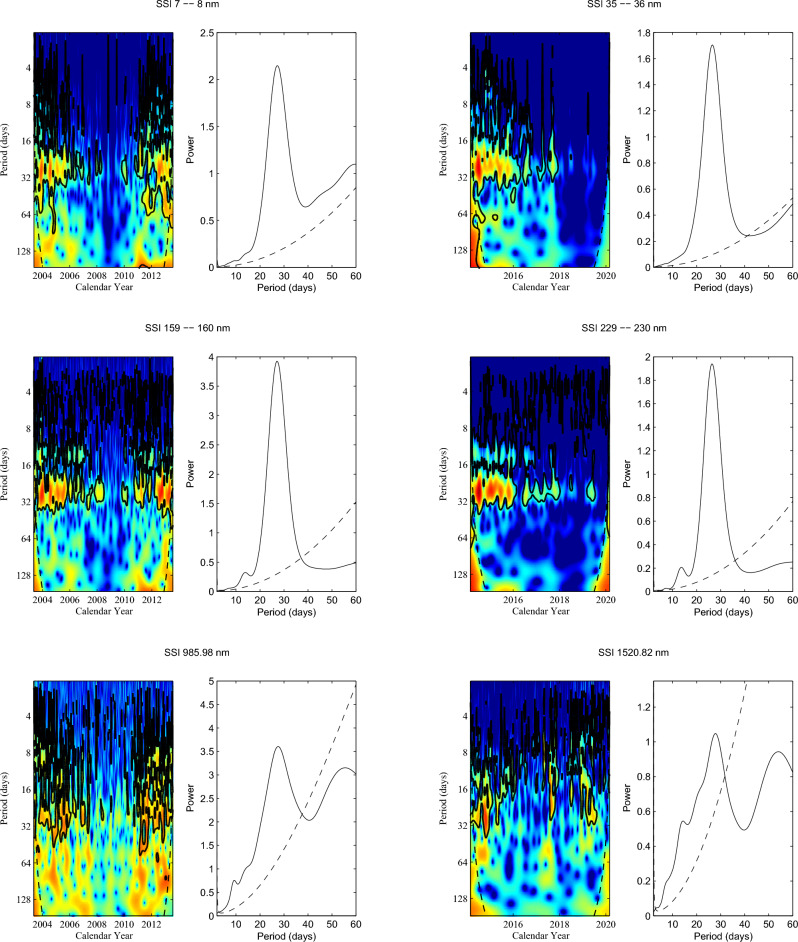
Continuous wavelet power spectra of the samples at six randomly selected bands of SSIs during the two time intervals considered. In each panel, the 95% confidence level and the cone of influence (COI), in which edge effects might distort the picture, are shown as the thick black contours and the black dashed line, respectively. Global wavelet power spectra (solid line) and the 95% confidence level (dashed line) are displayed in right column of each corresponding panel.

Codes of the CWT are derived from Grinsted et al.^33^. We use the CWT to analyze the six randomly selected bands of SSIs during the two time intervals considered, and the results are shown in Fig. 2. In this figure, the left column of each panel displays the continuous wavelet power spectra of a corresponding sample SSI, where the thick black contours indicate the 95% confidence level. Based on the local “components” of continuous wavelet power spectra, the time-averaged wavelet spectrum over the entire time interval considered, i.e. the global power spectra, is calculated and shown in the right column of each corresponding panel. As Fig. 2 shows, the continuous wavelet power spectra exhibit the localized oscillatory feature of the samples SSIs in the rotation period-scale range of 22–33 days. This clearly indicates that the rotation periods vary with time. The global power spectra determine that the dominant rotation periods for the six samples SSIs with wavelengths ranging from short to long are 27.4, 26.6, 27.0, 26.2, 27.8 and 27.8 days, respectively. In order to test the effect of these data gaps on the findings, we use anther three methods to fill the data gaps: (i) the data gaps are filled by the average of their neighboring values; (ii) the data gaps are filled by their preceeding neighbor observation; and (iii) the data gaps are filled by their following neighbor measurement. We find that the three methods and the linear interpolation are used to fill the gaps of the daily SSIs, almost the same results are obtained.

**Figure 3:**
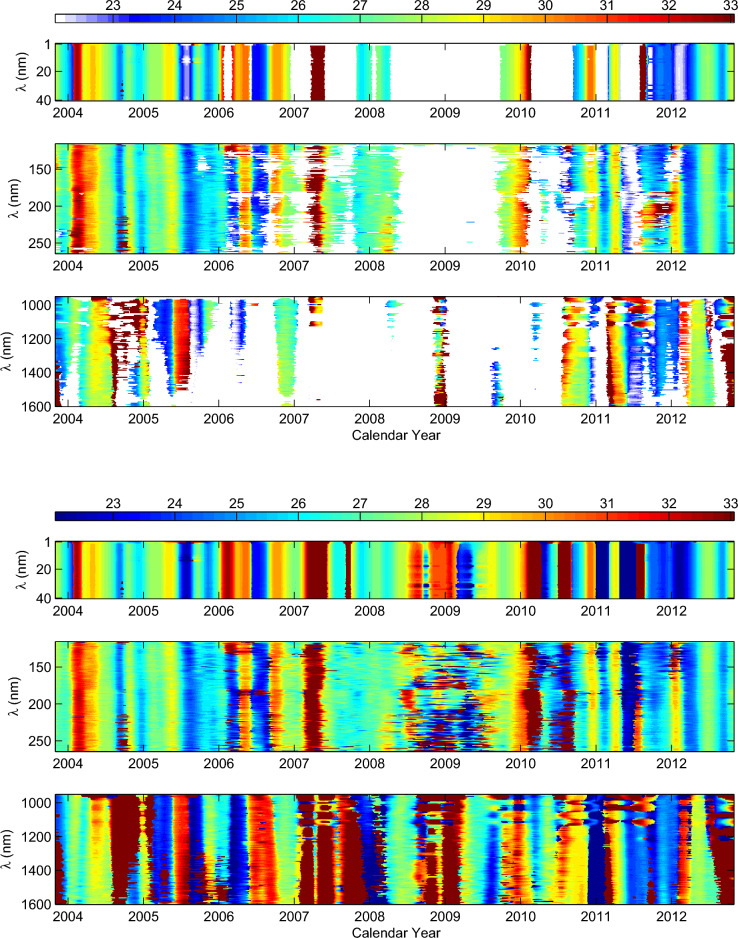
Top panel: temporal variation of the significant rotation periods for all SSIs considered that are during the time interval of 2003 April 14 to 2013 July 15. In order to ensure that the obtained results are the real rotation periods, 200 data points of local wavelet power spectra at the beginning and end are deleted. The values of rotation period are indicated by the color bay, and white points represent the no data points where the rotation periods of the corresponding SSIs are statistically insignificant. Bottom panel: same as the top panel, but showing the rotation periods for all SSIs without considering statistical significance.

**Figure 4:**
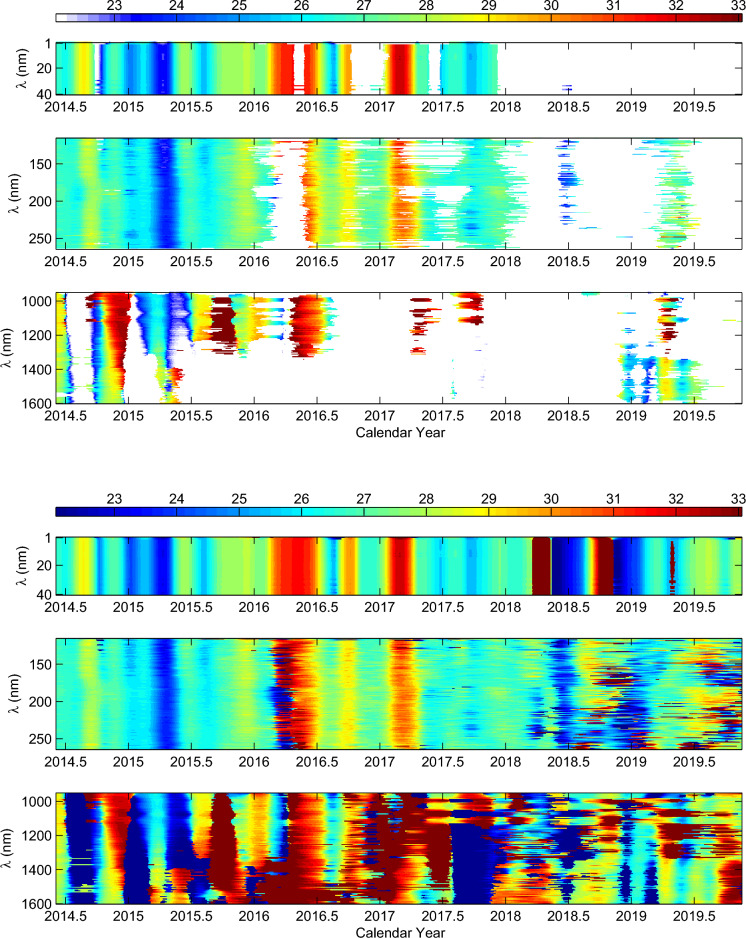
Same as Fig. 3, but for SSIs during the time interval of 2014 February 26 to 2020 February 25, and 100 data points of local wavelet power spectra at the beginning and end are deleted.

The local wavelet power spectra of the samples SSIs have the highest spectral power peaks at a certain day on the rotation timescales of 22–33 days. If these highest spectra power peaks are higher than their corresponding the 95% confidence level, the significant rotation period of the samples SSIs in each day during the time interval considered can be determined by using these highest spectral power peaks. In the same way, the significant rotation period of all SSIs in each day can be determined, respectively, during the two time intervals of 2003 April 14 to 2013 July 15 and 2014 February 26 to 2020 February 25. However, due to the fact that the SSIs are finite-length series, the local wavelet power spectra of the SSIs at the beginning and end may be artifacts. In order to ensure that the obtained results are the real rotation periods, we do not take into account the 200 data points of local wavelet power spectra at the beginning and end in time interval of former, such case also in time interval of latter but 100 data points at the beginning and end are not considered. The significant rotation periods for all SSIs in each day during the two time intervals considered are shown in Figs. 3 and 4, respectively. At the same time, the rotation periods for all SSIs in each day, without considering statistical significance, are also displayed in the two figures for comparison. In this scenario, some rotation periods for these SSIs are below the 95% confidence level. This may be attributed to the relatively weak rotation signal of the SSIs during a specific time interval as well as possible data gaps (accounting for only 2.0–2.7%). However, even though these periods are below the 95% confidence level, they may still exceed a certain confidence level and, to some extent, can exhibit the temporal variation of rotation for these SSIs on long timescales. In the subsequent analysis, we consider the significant rotation periods and the rotation periods without regard to statistical significance, respectively. The results obtained in both cases are used for mutual verification and complementation.

As the two figures show, both the significant rotation periods and the rotation periods without regard to statistical significance are all taken into account, the rotation periods for all SSIs vary with time and exist rotational modulation. For the SSIs at the spectral intervals 1–40 nm, the rotation periods exhibit consistent temporal evolution throughout the entire time interval considered (2003 April 14 to 2020 February 25). Similarly, from the beginning time to the first half of 2005 and from the second half of 2012 to the end of 2015, the temporal evolution of the rotation periods for the SSIs at the spectral intervals 116–264 nm is also almost consistent, and such temporal evolution is almost consistent with the temporal evolution of the rotation periods for the SSIs at the spectral intervals 1–40 nm in these time intervals. However, from the second half of 2005 to the first half of 2012 and from 2016 to the ending time, the temporal evolution of the rotation periods for the SSIs at the spectral intervals 116–264 nm shows a coexistence of consistency and discrepancy. The consistency of temporal evolution of rotation periods is consistent with the temporal evolution of the rotation periods for the SSIs at the spectral intervals 1–40 nm, while the discrepancy of temporal evolution varies with time. This seems to be related to the solar activity, and the maximum discrepancy occurs during the minimum of solar cycle. For the SSIs at the spectral intervals 950–1600 nm, the temporal evolution of the rotation periods shows relatively large discrepancies in some regions of the two figures, but the overall trend of the temporal evolution remains generally consistent throughout the entire time interval considered. However, the temporal variation of the rotation periods for these SSIs is almost different with that of rotation for the SSIs at the spectra intervals 1–40 and 116–264 nm in most time of the entire time interval considered. Additionally, the two figures also indicate that the rotation periods of SSIs at the spectra intervals 950–1600 nm are generally longer than those of SSIs at the spectra intervals 1–40 and 116–264 nm. Nevertheless, in some times, the rotation periods of SSIs at spectra intervals 950–1600 are shorter than/or very close to the rotation periods of SSIs at spectra intervals 1–40 and 116–264 nm.

## Relation of the coronal rotation with the underlying photosphere

**Figure 5:**
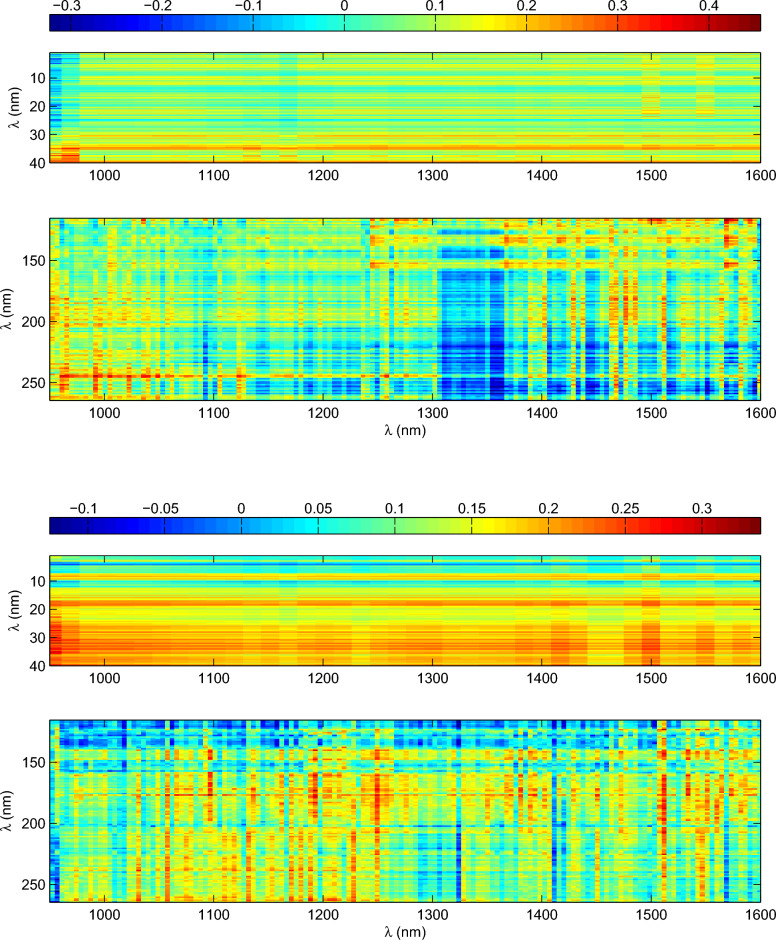
Top panel: correlation coefficients of the significant rotation periods for the SSIs at the spectral intervals 1–40 and 116–264 nm, respectively, with that for the SSIs at the spectra intervals 950–1600 nm. Bottom panel: same as the top panel, but for the rotation periods of all SSIs without regard to statistical significance. In each panel, the correlation coefficients are indicated by the color bay.

**Figure 6:**
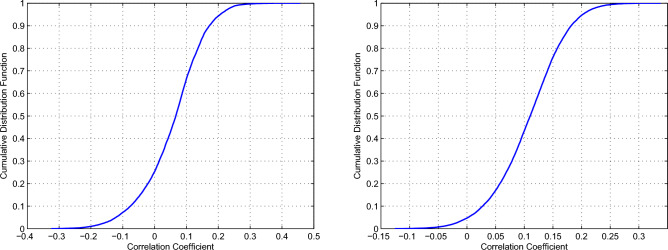
Left panel: the cumulative probability distribution of correlation coefficients of the significant rotation periods for the SSIs at the spectral intervals 1–40 and 116–264 nm, respectively, with that for the SSIs at the spectra intervals 950–1600 nm. Right panel: same as the left panel, but for the rotation periods of all SSIs without regard to statistical significance.

In this study, correlation is performed by Pearson correlation analysis. Daily rotation periods for all SSIs in the entire time interval considered (2003 April 14 to 2020 February 25) are used to calculate correlation coefficients between the rotation periods of SSIs that form in the corona and those formed at the bottom of the photosphere. Regarding the rotation periods without considering statistical significance, according to the results in Figs. 3 and 4, we can obtain a matrix of daily rotation periods, and these rotation periods for all SSIs correspond to each other in time. Thus, we can calculate the correlation coefficients between the rotation periods of SSIs at the spectral intervals 1–40 nm and 116–264 nm, respectively, and the rotation periods of SSIs at the spectral intervals 950–1600 nm. The obtained correlation coefficients form a matrix with 23247 values, which is shown in Fig. 5. For the significant rotation periods, we can also obtain a similar matrix of daily rotation periods for all SSIs considered. However, the daily rotation periods for SSIs are statistically insignificant in some times, resulting in null values in this matrix. Therefore, the significant rotation periods for all SSIs do not completely correspond to each other in time. In principle, only by finding the corresponding significant rotation periods in time between two sets of data from this similar matrix can we calculate the correlation coefficient between them. Thus, for each correlation coefficient calculation, the null values in two selected time series are deleted to ensure that daily rotation periods in the two time series correspond to each other in time. The correlation coefficients of the significant rotation periods for the SSI at the spectra intervals 1–40 and 116–264, respectively, with that for the SSIs at the spectra intervals 950–1600 nm are calculated. The results are also shown in Fig. 5. Meanwhile, we further calculate the cumulative probability distribution of correlation coefficients for the two scenarios shown in Fig. 5, respectively. Taking the correlation coefficients shown in top panel of Fig. 5 as an example, these correlation coefficients can be considered as a set S. For a certain correlation coefficient $\mu$ ($\mu$ $\in$S), we can calculate the probability mass function $f(\mu )$, then the cumulative distribution function is given by F(x)=$\sum _{\mu \le x}f(\mu )$. The obtained results are shown in Fig. 6, which can further help understand the distribution of correlation coefficients.

Whether considering the significant rotation periods or the rotation periods without regard to statistical significance, both figures indicate that most of the correlation coefficient values are small and relatively concentrated. When considering the significant rotation periods, there are 23247 correlation coefficient values. The minimum and maximum absolute values of these correlation coefficients are $1.162\times 10^{-5}$ and 0.4576, respectively. Among these correlation coefficients, absolute values less than 0.2 account for 93.27%, and absolute values less than 0.25 are as high as 98.45%. Similar results also present when the rotation periods of all SSIs without regard to statistical significance are taken into account. The maximum absolute value of the correlation coefficient is 0.3382, and the minimum absolute value is $2.7\times 10^{-5}$. Absolute values less than 0.2 account for 93.47%, and absolute values less than 0.25 are up to 99.2%. Thus, these results based on the correlation analysis indicate that the rotation variation of the SSIs that form in the corona is weakly related to those formed at the bottom of the photosphere.

**Figure 7:**
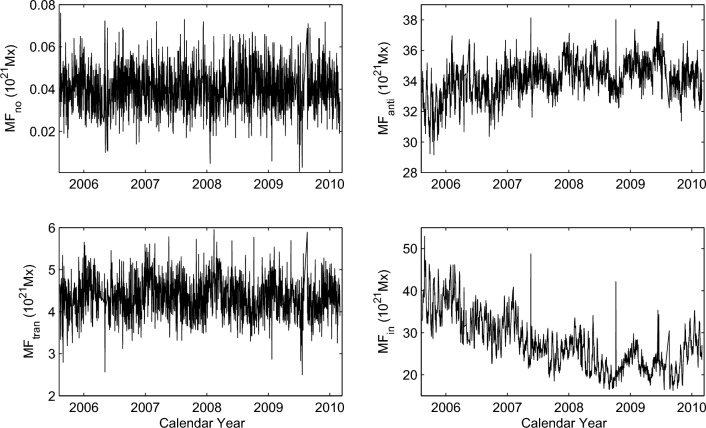
Small-scale magnetic elements of the daily $MF_{no}$, $MF_{anti}$, $MF_{tran}$ and $MF_{in}$ during the time interval of 2005 August 11 to 2010 February 28.

**Figure 8:**
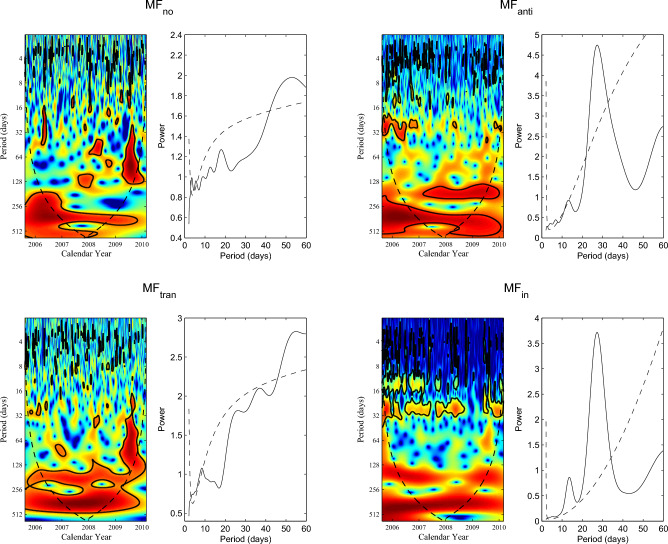
Continuous wavelet power spectra of the daily $MF_{no}$, $MF_{anti}$, $MF_{tran}$ and $MF_{in}$ during the time interval of 2005 August 11 to 2010 February 28. In each panel, the 95% confidence level and the cone of influence (COI) in which edge effects might distort the picture are shown as the thick black contours and the black dashed line, respectively. Global wavelet power spectra (solid line) and the 95% confidence level (dashed line) are displayed in right column of each corresponding panel.

**Figure 9:**
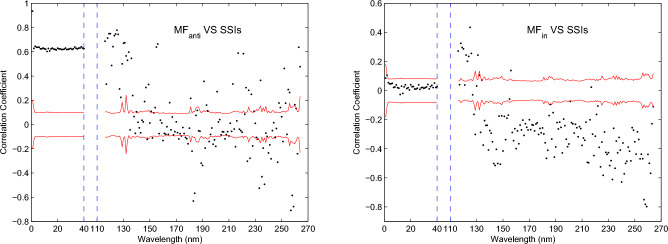
Left panel: correlation coefficients of the significant rotation periods for daily $MF_{anti}$, respectively, with that for the SSIs at the spectral intervals 1–40 and 116–264 nm. Right panel: same as the left panel, but for the rotation periods for daily $MF_{in}$, respectively, with that for the SSIs at the spectral intervals 1–40 and 116–264 nm. In each panel, the correlation coefficients are shown by black dots, and the two red lines indicates the 95% confidence level. The correlation coefficients that are located between the two red lines are of statistical insignificance.

## Relation of the coronal rotation with the small-scale magnetic elements

The 5 minute averaged full-disk magnetograms measured by the MDI/SOHO with a pixel size of $2^{\prime \prime }$ are used to extracted the daily $MF_{no}$, $MF_{anti}$, $MF_{tran}$ and $MF_{in}$ in solar cycle 23. On each day, one averaged magnetogram is extracted, and a total of 3764 magnetograms is selected during the time interval of 1996 September to 2010 February. All selected magnetograms are smoothed with a boxcar function to further reduce the noise level, and the magnetic flux of pixels on the magnetograms is corrected according to its heliocentric angle of each pixel, because the MDI instrument observed the line-of-sight magnetic filed, which is a projection of the intrinsic flux density. Due to the low magnetic sensitivity and spatial resolution as well as high magnetic noise level, only a few magnetic signals are observed in pixels with heliocentric angles larger than $60^{\circ }$. Thus, only the pixels with heliocentric angles smaller than $60^{\circ }$ are selected. Only these areas larger than $9\times 9$ pixels and with a magnetic flux density of at least 15 Mx $cm^{2}$ are considered as active regions, and those relatively small areas with weak magnetic fields on the magnetograms are defined as small-scales magnetic elements. More detailed data extraction methods were given in Jin et al.^26^.

Then, the four categories of flux data, i.e. daily $MF_{no}$, $MF_{anti}$, $MF_{tran}$ and $MF_{in}$, can be obtained in the time interval of 1996 September to 2010 February. There, the classification of small-scale magnetic elements is based on the correlation coefficients between the sunspot number and network elements, boundary values of magnetic flux of the $MF_{no}$, $MF_{anti}$, $MF_{tran}$ and $MF_{in}$ may have very small deviations. However, our subsequent analysis mainly focuses on analyzing the relation of small-scales magnetic elements with coronal rotation, which is indicated by the rotation of SSIs at the spectral intervals 1–40 and 116–264 nm. Hence, the influence of minor deviations in magnetic flux boundary values on the correlation analysis of extensive data sets is almost negligible. During this time interval, there are a total of 4929 days, but only 3764 days of magnetic flux data were recorded. It is evident that there are numerous data gaps in the time series for all four categories of magnetic flux. However, from 2005 August 11 to 2010 February 28, there were relatively few and randomly distributed data gaps in the magnetic flux data records. The continuous data gaps in these time series were generally shorter than 5 days. The four categories of magnetic flux during the time interval of 2005 August 11 to 2010 February 28 are presented in Fig. 7, which can be employed to explore the relation of the coronal rotation with the small-scale magnetic elements. The method of the linear interpolation is utilized to interpolate the data gaps of the four categories of flux data. Subsequently, the CWT is once again employed to analyze the daily $MF_{no}$, $MF_{anti}$, $MF_{tran}$ and $MF_{in}$. The results are shown in Fig. 8.

As Fig. 8 shows, the wavelet power spectra of the daily $MF_{no}$ and $MF_{tran}$ reveal that only a few small regions exhibit statistically significant oscillations on the timescales of a solar rotation period. The corresponding global power spectra indicate that these two data sets of magnetic flux data have no statistically significant rotation period. Contrary to that, continuous regions with statistically significant oscillations on the timescales of a solar rotation period can be found on the wavelet power spectra of both daily $MF_{anti}$ and $MF_{in}$. These two categories of small-scale magnetic elements exhibit statistically significant rotation periods of 26.6 and 27.0 days, respectively. The results obtained in this study agree well with the findings in Xu et al.^38^, where authors employed the Lomb-Scargle method to detect the rotation periods of these categories of small-scale magnetic elements.

Based on the CWT analysis, the significant rotation periods of $MF_{anti}$ and $MF_{in}$ on each day during the time interval of 2005 August 11 to 2010 February 28 can be determined. The 100 data points of local wavelet power spectra at the beginning and end for the two categories of flux data are not considered, as the two data sets are finite-length series and their local wavelet power spectra at the beginning and end may be artifacts. The significant rotation periods of $MF_{anti}$ and $MF_{in}$ can be used to investigate the relation of the coronal rotation with the small-scale magnetic elements. It’s worth noting that there are some null values in the daily significant rotation periods of SSIs formed in the corona, as well as in $MF_{anti}$ and $MF_{in}$ during the time interval of 2005 August 11 to 2010 February 28. We do not extract the significant rotation periods of $MF_{no}$ and $MF_{tran}$ on each day, because their local wavelet power spectra shown in Fig. 8 indicate that there is almost no significant rotation period in this time interval, making it unsuitable for further analysis. We calculate the correlation coefficients between the significant rotation periods of $MF_{anti}$ and those of SSIs at the spectral intervals 1-40 and 116-264 nm, respectively. To the calculation of each correlation coefficient, the null values in two selected time series, i.e. in daily rotation periods of $MF_{anti}$ and SSIs, are deleted to ensure that daily rotation periods in the two time series correspond to each other in time. Similarly, the correlation coefficients of the significant rotation periods for $MF_{in}$, respectively, with those for SSIs at the spectral intervals 1–40 and 116–264 nm are also determined. Meanwhile, the critical values at the 95% confidence level for each correlation coefficient are also given based on the actual number of significant rotation periods used for calculation. All results are shown in Fig. 9.

For SSIs with wavelengths spanning 1–40 nm, Fig. 9 clearly shows that all correlation coefficients between the significant rotation periods of $MF_{anti}$ and those of SSIs are bigger than 0.6 and above the 95% confidence level, while all correlation coefficients between the significant rotation periods of $MF_{in}$ and those of SSIs are very small (absolute values less than 0.1) and have no statistical significance. The variations of rotation for these SSIs are highly positively correlated with that of rotation for $MF_{anti}$, but should be weakly related to that of rotation for $MF_{in}$. Furthermore, the pattern of the temporal variation of rotation (Figs. 3 and 4) shows consistent temporal evolution, which hints that the variations of rotation for these SSIs should be derived from the same physical origin. Therefore, the temporal variation of rotation for the SSIs with wavelengths spanning 1–40 nm are mainly dominated by the small-scale magnetic elements of $MF_{anti}$ during this time interval. For the SSIs at the spectral intervals 116-264 nm, the correlation coefficients between the significant rotation periods of $MF_{anti}$ and those of SSIs are dispersively distributed. Among these correlation coefficients, 52 values show statistically significant positive correlation, 40 values indicate statistically significant negative correlation, and 57 values are of statistical insignificance. Therefore, the relationship between the temporal variation of rotation for SSIs with wavelengths spanning 116-264 nm and the small-scale magnetic elements of $MF_{anti}$ is complex, but it can be determined that the small-scale magnetic elements of $MF_{anti}$ still contributes to the temporal variation of rotation for these SSIs. For these correlation coefficients between the significant rotation periods of $MF_{in}$ and those of SSIs at the spectral intervals 116-264 nm, 8 of them are small values that are of statistical insignificance, only 12 values indicate statistically significant positive correlation, and 129 values, accounting for 86.6% of the total number, show statistically significant negative correlation. So the temporal variation of rotation for these SSIs with wavelength spanning 116-264 nm is also related to that of rotation for the small-scale magnetic elements of $MF_{in}$, and in general, the relationship between them is negative correlation.

## Conclusions and discussion

In this work, all of the obtained rotation periods have no relation to differential and can be seen as average over latitudes. As in early studies, the rotation is investigated from a global point of view^21,38,39^. The rotation periods for SSIs formed in the corona should represent that of the coronal plasma atmosphere^21^. Because the corona is highly structured and contains many substantial magnetic loops on different spatial scales. Due to Lorentz force derived from magnetic field and the low $\beta$ value in corona, coronal emission with strong spatial heterogeneity is determined by the magnetic structure^40,41^. The rotation periods for SSIs formed at the underlying photosphere are that of the solar atmosphere at the underlying photosphere modulated by large-scale magnetic structures^21^. Since the temperature of sunspots is lower than that of their background photosphere, sunspots look like dark structures and can be considered as sunspot blocking in the theory of solar spectral radiation. Thus, sunspots decrease both SSIs formed in the photosphere and total solar irradiance^42,43^. Additionally, the small-scale magnetic elements can be considered as small-scale brightening, which should increase these SSIs formed at the underlying photosphere. However, high-resolution observations indicate that the small-scale magnetic activity is ubiquitous on the solar surface, so the rotation periods of these SSIs formed at the underlying photosphere are not significantly modulated by the ubiquitous small-scale magnetic activity.

Temporal variation in the rotation of the corona and the underlying photosphere shows that the rotation periods of SSIs at the spectra intervals 950-1600 nm are generally longer than those of SSIs at the spectral intervals 1–40 and 116–264 nm. This actually indicates that the coronal atmosphere generally rotates faster than the underlying photosphere. Similarly, Mancuso & Giordano^44^ and Mancuso et al.^8^ found that the corona obviously rotates faster than the magnetic structures in the photosphere. The average rotation period of the equatorial region of EUV corona is obviously shorter than that of the photosphere and chromosphere^17^. The radio corona also rotates faster than photosphere to some extent^2,7,9,13,45^. However, Figs. 3 and 4 also indicate that the rotation of underlying photosphere are faster than/or very close to that of coronal atmosphere in some time intervals. This finding does somewhat provide reasonable explanations for another seemingly contradictory result: why some studies, based on the analysis of specific X-ray and ultraviolet spectral lines, have found that the coronal rotation may be comparable to, or even slower than, the rotation of the underlying photosphere^5,15,46–48^. Actually, the result in these early studies does not conflict with our findings. If these spectral lines used in early studies are within/or contain the special time interval in which the rotation for these SSIs formed in the corona is slower than/or very close to that of the underlying photosphere, the seemingly contradictory result may be found. On the whole, the temporal variation of rotation for the coronal atmosphere is almost different with that of rotation for the solar atmosphere at the underlying photosphere modulated by large-scale magnetic structures. Furthermore, correlation analysis indicates that the rotation variation of the SSIs that form in the corona is weakly related to that of the SSIs formed at the bottom of the photosphere. These results directly confirm that the temporal variation of rotation for the coronal atmosphere is weakly related to the large-scale magnetic structures at the underlying photosphere.

Wavelet analysis suggests that both $MF_{no}$ and $MF_{tran}$ have no periodicity on the timescales of a rotation period. The flux of the two categories of small-scale magnetic elements accounts for only 0.05% and 9.08% of the total magnetic flux of small-scale magnetic elements, respectively^26^. These results hint that the temporal variation of rotation for the coronal atmosphere should not be related to the small-scale magnetic elements of $MF_{no}$ and $MF_{tran}$. However, relation of the coronal rotation with the small-scale magnetic elements indicates that the temporal variation of rotation for the SSIs with wavelengths spanning 1-40 nm are mainly dominated by the small-scale magnetic elements of $MF_{anti}$ during time interval of 2005 August 11 to 2010 February 28. This result is found in a relatively short time interval. In the entire time interval considered, which contains all phases of solar cycle, the rotation periods for the SSIs with wavelength spanning 1–40 nm exhibit consistent temporal variation. If there is other factor effect on the variation of rotation for these SSIs, the consistent temporal variation of rotation is bound to be disrupted. The pattern of the temporal variation of rotation should display the coexistence of consistency and discrepancy, like the temporal variation of rotation periods for SSIs with wavelengths spanning 116–264 nm in the time interval of the second half of 2005 to 2012. However, such pattern of the temporal variation of rotation for the SSIs with wavelength spanning 1–40 nm does not appear during the entire time interval considered. Thus, the consistent temporal variation of rotation for the SSIs with wavelengths spanning 1–40 nm is mainly dominated by the small–scale magnetic elements of $MF_{anti}$ in all phases of solar activity cycle.

From beginning time to first half of 2005 and from second half of 2012 to end of 2015, the two time intervals, respectively, correspond to the first half of the declining phase of solar cycle 23, during which solar activity can almost match the maximum of solar cycle 24^49^, and the maximum of solar cycle 24. The temporal evolution of the rotation periods for SSIs at the spectral intervals 116–264 nm is almost consistent with the temporal evolution of the rotation periods for SSIs at the spectral intervals 1–40 nm, suggesting that the variations of rotation for these SSIs during the epochs of strong solar activity are mainly related to the small-scale magnetic elements of $MF_{anti}$. However, for these SSIs during the time interval of 2005 August 11 to 2010 February 28, correlation analysis indicates that the variations of rotation are closely correlation with those of rotation for both $MF_{anti}$ and $MF_{in}$, and the pattern of the temporal variation of rotation (Fig. 3) shows the coexistence of consistency and discrepancy. In this time interval, solar activity weakened as the solar cycle advanced, ending the solar cycle 23 in the years of 2008.958, and then entering the minimum of solar cycle 24. Correspondingly, the consistency in the temporal variation of rotation for the SSIs with wavelengths spanning 116-264 nm is decreasing but always exists to some extent, and this consistency is also consistent with the temporal evolution of rotation periods for SSIs in the spectral range of 1–40 nm. Therefore, the small-scale magnetic elements of $MF_{anti}$ still contribute to the temporal variations of rotation for these SSIs. On the other hand, the discrepancy in temporal variation of rotation for the SSIs with wavelengths spanning 116–264 nm is more and more, reaching maximum during the minimum of solar cycle 24. This suggests that the small-scale magnetic elements of $MF_{in}$ have a significant effect on the temporal variation of rotation for these SSIs. The weaker the solar activity, the stronger the effect would be, and the maximum effect is reached during the solar minimum (negative correlation). After 2009, with the advance of solar cycle 24, solar activity became increasingly stronger. The discrepancy in the temporal variation of rotation for the SSIs with wavelengths spanning 116–264 nm increasingly decrease, and it changes from coexistence of consistency and discrepancy to consistency near the solar maximum. Correspondingly, the effect of the small-scale magnetic elements of $MF_{in}$ on the temporal variation of rotation for these SSIs is weaker and weaker. Finally, it is mainly dominated by the small-scale magnetic elements of $MF_{anti}$ once again near the solar maximum. Furthermore, the above results are further confirmed within the time interval of 2016-2020.

To sum up, during the solar maximum, the temporal variation of rotation for all SSIs formed in the corona, i.e. rotation of the coronal plasma atmosphere, is mainly dominated by the small-scale magnetic elements of $MF_{anti}$. While during the epochs of relatively weak solar activity, it is controlled by the joint effect of the small-scale magnetic elements of both $MF_{anti}$ and $MF_{in}$. The weaker the solar activity, the stronger the effect of $MF_{in}$ would be. Mancuso et al.^8^ found that the rotation of the quiet corona is connected to the intermediate-scale magnetic structures rooted near 0.99$R_{\odot }$. Li et al.^21^ reported that the rotation of the coronal plasma atmosphere is mainly modulated by ubiquitous small-scale magnetic activity. These recent studies support our results to some extent. However, there is a question as to why the small-scale magnetic elements of $MF_{in}$ have a significant effect on the temporal variation of rotation for the coronal plasma atmosphere only during the epochs of relatively weak solar activity.

It is widely accepted that solar violent activities and slow variations can be explained by magnetic activities, and solar activities at different altitudes in the solar atmosphere are linked to the different categories (scales) of magnetic fields^40,50–52^. Similarly, Li et al.^53^ further demonstrated that the magnetic activities of different scales can extend to different heights from the photosphere to the upper atmosphere. Our finding shows that the variation of rotation for coronal plasma is linked to the small-scale magnetic elements of both $MF_{anti}$ and $MF_{in}$, and is regulated by them. Thus, the ubiquitous small-scale magnetic elements of $MF_{anti}$ and $MF_{in}$ extend to the corona and guide coronal plasma in the structures. The rotation period of the coronal plasma atmosphere should reflect that of mall-scale magnetic activity of $MF_{anti}$ and $MF_{in}$. It is known that large sunspots rotate slower than small-scale magnetic structures^10,21,38^, and thus the small-scale magnetic elements of $MF_{anti}$ and $MF_{in}$ cause the coronal plasma atmosphere to rotate faster than the underlying photosphere. Li et al.^21^ reported that the coronal atmosphere abnormally rotates faster than the underlying photosphere, considered to be another big question, as the big question of abnormal high temperatures in the corona. Our finding directly answers this big question based on the relationship between coronal rotation and magnetic field structures. On the other hand, during the solar maximum, the small-scale magnetic elements of $MF_{in}$ almost have no effect on the temporal variation of rotation for corona plasma. In that way the magnetic flux of $MF_{in}$ during this time interval should not extend to the corona.

The key issue is why the stronger magnetic flux of $MF_{in}$ during the solar maximum does not extent to the corona. It is known that the ubiquitous small-scale magnetic elements outside of active regions cover everywhere on the Sun^22,54^. High-resolution observations indicate that the ubiquitous small-scale magnetic elements are derived from several sources: debris of decayed active regions, small-scale flux emergence in the ephemeral regions, coalescence of intra-network elements, and products of dynamic interaction among different scales of magnetic activities^22,26^. Vögler & Schüssler^55^ demonstrated that the small-scale magnetic elements at the larger flux end of the flux spectrum are likely to be the debris of decayed active regions, and, of course, are in phase with the sunspot cycle. Therefore, the debris of decayed active regions are one of the main components of the small-scale magnetic elements in $MF_{in}$. However, even in the “huge” minimum of solar cycle 24 with either no or only a few sunspots visible on the disk, the magnetic flux of $MF_{in}$ also had a significantly large value, reaching almost one third of that during the maximum of solar cycle 23^26^. Furthermore, the formation of network elements is attributed to the debris of decayed active regions and the small-scale emerging bipoles. Compared to the intra-network elements, the network elements at the boundaries of super-granulation cells are the stronger magnetic elements^25^. Hence, the strong magnetic elements derived from the small-scale flux emergence also constitute important components of the small-scale magnetic elements of $MF_{in}$.

During the epochs of the relatively weak solar activity, for instance, from August 2005 to February 2010, as the solar cycle progressed, solar activity became increasingly weaker. As a result, the components derived from the debris of decayed active regions in $MF_{in}$ decreased, whereas those originated from small-scale flux emergence potentially increased. This is because the latter components are increasingly less affected by dynamic interaction between them and active regions, such as cancellations and merging of magnetic flux. Especially during the solar minimum, the small-scale magnetic elements of $MF_{in}$ primarily originate from small-scale flux emergence, owing to the absence or limited presence of sunspots on the disk. Consequently, it is quite certain that the components derived from the small-scale flux emergence in $MF_{in}$ can extend to the corona and have the greatest effect on the temporal variation of rotation for corona plasma during the solar minimum.

During solar maximum, substantial debris of decayed active regions contribute to the increased magnetic flux of $MF_{in}$. Due to a quantity of sunspots on the solar disk, the smaller magnetic components derived from the small-scale flux emergence in $MF_{in}$ have a greater opportunity to encounter active regions and their fragments. The encounter between those elements of opposite polarity results in flux cancellations, which ultimately leads to the loss of smaller magnetic elements and a diffusion of sunspot flux. The encounter of the same polarity, however, leads to a merging of magnetic flux, which causes those smaller magnetic elements to merge into the flux related to sunspots. Such dynamic interaction is sufficient during the solar maximum, resulting in almost non-existent the smaller magnetic component derived from the small-scale flux emergence in $MF_{in}$. But actually, these smaller magnetic components have been confirmed to have a significant effect on the temporal variation of rotation for corona plasma. Thus, during the solar maximum, the small-scale magnetic elements of $MF_{in}$ primarily originate from debris of decayed active regions, owing to the presence of sufficient sunspots on the solar disk. At the same time, based on the anterior analysis, the presence of such composition in $MF_{in}$ indicates that these components, derived from the debris of decayed active regions, have no effect on the temporal variation of rotation for corona plasma, and so should not extend to the corona. As height increases from the photosphere to the upper atmosphere, the solar magnetic fields of different scales vary to more and more horizontal^56^, and the magnetic activities of different scales extend to different height in the solar atmosphere^53^. According to our findings, the magnetic fields derived from the debris of decayed active regions should become horizontal at a certain height below the corona layer, because such a scenario is in accordance with the relationship between the temporal variation of coronal rotation and the small-scale magnetic elements of $MF_{in}$. This can also explain why the small-scale magnetic elements of $MF_{in}$ during the solar maximum, which are mainly derived from the debris of decayed active regions, have no effect on the temporal variation of rotation for corona plasma. Additionally, the small-scale magnetic elements of $MF_{anti}$ are in anti-phase with sunspot cycle, but the magnetic flux of $MF_{anti}$ still has a considerable value during the solar maximum^26^. Therefore, these magnetic elements have a significant effect on the temporal variation of rotation for corona plasma in all phases of solar activity cycle.

In summary, this study reveals the contributions of different magnetic structures to the temporal variation of the rotation for the coronal atmosphere during different phases of the solar cycle. During the solar maximum, the temporal variation of rotation for the coronal plasma atmosphere is mainly dominated by the small-scale magnetic elements of $MF_{anti}$; whereas during the epochs of the relatively weak solar activity, it is controlled by the joint effect of the small-scale magnetic elements of both $MF_{anti}$ and $MF_{in}$. The weaker the solar activity, the stronger the effect of $MF_{in}$ would be. Furthermore, our findings present an explanation for the inconsistent results for the coronal rotation issue among the previous studies, and also reveal the reason why the coronal atmosphere rotates faster than the lower photosphere.

## Data Availability

(1) Daily SSIs can be downloaded from web site: http://lasp.colorado.edu/home/sorce/data/. (2) The four categories of flux data (daily $MF_{no}$, $MF_{anti}$, $MF_{tran}$ and $MF_{in}$) come from Dr. Jin Chun-Lan, and data requests should be sent to cljin@nao.cas.cn.
